# The Ecto-Endodermal Boundary in the Oral and Pharyngeal Mucosa: A Narrative Review

**DOI:** 10.3390/life16081258

**Published:** 2026-07-30

**Authors:** Rogier Schipperheijn, Frederik G. Dikkers, Bernadette S. de Bakker

**Affiliations:** 1 Department of Obstetrics and Gynecology, Amsterdam UMC Location University of Amsterdam, Meibergdreef 9, 1105 AZ Amsterdam, The Netherlands; r.schipperheijn@amsterdamumc.nl; 2 Amsterdam Reproduction and Development Research Institute, 1105 AZ Amsterdam, The Netherlands; 3 Department of Otorhinolaryngology, Amsterdam UMC Location University of Amsterdam, 1007 MB Amsterdam, The Netherlands; f.g.dikkers@amsterdamumc.nl; 4 Department of Otorhinolaryngology, University Medical Center Groningen, University of Groningen, 9700 RB Groningen, The Netherlands; 5 Department of Pediatric Surgery, University Medical Center Rotterdam, Erasmus MC—Sophia Children’s Hospital, 3000 CB Rotterdam, The Netherlands

**Keywords:** human embryology, embryonic development, human mouth, ectoderm, endoderm, oral cancer, head and neck cancer

## Abstract

**Introduction:** The ecto-endodermal boundary in the human oral cavity remains debated. Rather than a distinct line, this transition forms a complex interface, especially in the developing mouth and pharynx. This ambiguity is clinically relevant, as, for example, HPV-induced tumors often arise where ecto- and endoderm meet. The boundary is generally placed at the posterior third of the tongue and behind the uvula, though its developmental basis remains unclear. This review summarizes the literature on the embryological development of the mouth and pharynx to trace origins and interactions of ectodermal and endodermal derivatives and identify potential tumor initiation regions. **Methods:** PubMed articles on oral structures were reviewed to approximate the ecto-endodermal border. **Results:** Teeth originate from ectoderm. The origin of salivary glands and papillae depends on their location. No study reports were found for the tonsils, incisive papilla, tubarial glands, and faucial pillars. **Conclusions:** A clear educational image of the origin of the structures of the human mouth is made to help identify regions of risk for oral cancer.

## 1. Introduction

**Figure 1:**
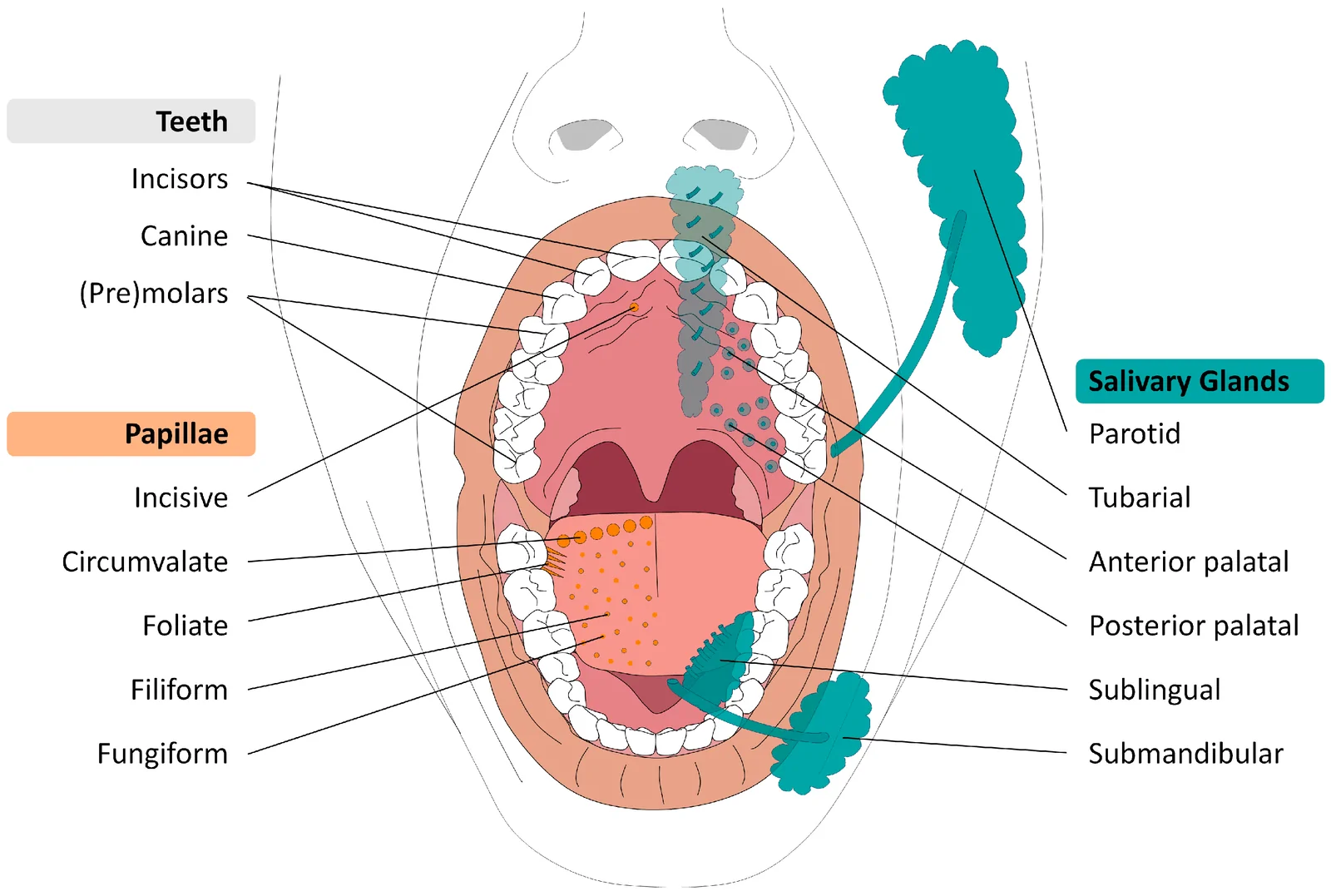
**Anatomy of the oral cavity.** Structures of the oral region are illustrated. Teeth are shown in white, papillae are shown in orange, and salivary glands are shown in turquoise.

HPV-induced cancers are preferentially located in parts of the human body where ecto- and endoderm are connected, such as the cervix, anus and the transition area between oral cavity and pharynx [1]. As opposed to the cervix and anus, the border between the ecto- and endoderm is still unclear in the oral region [2]. It is estimated that 9.5% of all tumor diagnoses involve the head and neck region worldwide, making it one of the ten most prevalent cancer types [3,4]. Within the oropharyngeal cavity, the regions where oral tumors occur the most are the tongue base and tonsils [5,6,7]. The transition between layers of different epithelial origins might play an important role in the development of tumors in the regions infected by HPV [8]. As HPV-induced tumors develop on the border between epithelial layers, for example, in the larynx (recurrent respiratory papillomatosis), pharynx, and cervix, understanding the embryonic development of these structures is relevant, especially for unknown primary tumors. Currently, the precise location of the border between ectoderm and endoderm in the oral cavity and pharynx remains controversial. Understanding this boundary is clinically significant, as it can reveal regions where cancer may preferentially develop following HPV infection, revealing locations at risk for currently unknown primary tumors (up to 5% of head and neck cancer cases) [9]. Our hypothesis is that a thorough knowledge of the embryonic origin of oropharyngeal structures enhances our understanding of the etiology of oral tumors. Through this review, we aim to describe the transition between ectoderm and endoderm in the mucosa of the head and neck, with a focus on the epithelial origin of various structures during embryology (Figure 1). By examining research on the development of teeth, salivary glands, tonsils, faucial pillars, and papillae, we seek to provide a more precise location of this critical border.

### Embryonic Development

**Figure 2:**
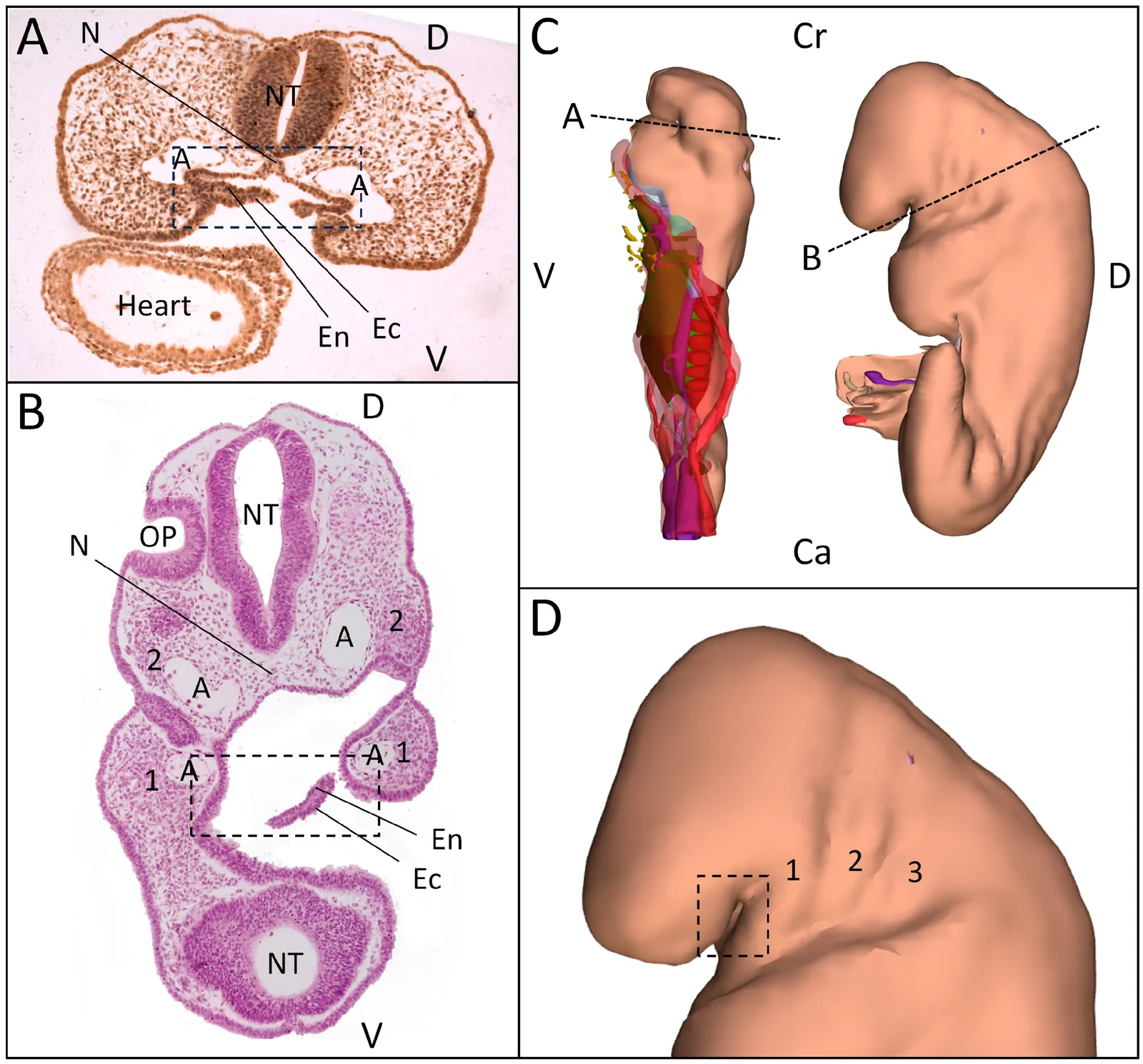
**Human embryos of Carnegie stage 11 and 12 showing the oropharyngeal membrane.** (**A**) Histological section of a Carnegie stage 11 human embryo, specimen nr. 6784 (23–26 days of development, 2.5 mm CRL). The square box indicates the location of the partly ruptured oropharyngeal membrane. (**B**) Carnegie stage 12 human embryo, specimen nr. 8505A, 26–30 days, 2.9 mm CRL. The square box indicates the remnant of the oropharyngeal membrane. Note how the endoderm and ectoderm are directly in contact, without mesoderm in between. (**C**) 3D reconstructions of the Carnegie stage 11 and 12 embryos. The dotted lines indicate the level of the sections in A and B. (**D**) 3D reconstruction of the head of the Carnegie stage 12 embryo, with emphasis on the location of the future mouth (stomodeum) and the first three pharyngeal arches. Abbreviations: 1/2/3 = first/second/third pharyngeal arch; A = artery; Ca = caudal; Cr = cranial; D = dorsal; Ec = ectoderm; En = endoderm; N = notochord; NT = neural tube; OP = otic pit; V = ventral. Images based on the 3D Embryo Atlas [8], with permission by Dr. de Bakker of Amsterdam UMC.

During embryonic development, the human oral cavity develops via the invagination of ectodermal tissue in the cranial region [10]. The oral cavity forms the passage between the outside and inside of the body, which originate from, respectively, the ecto- and endoderm. Ectodermal epithelium gives rise to the nervous system and skin. Endodermal epithelium classically contributes to the inner lining of organs such as the gut and the lungs [11]. These germ layers are directly connected in the cranial region, referred to as the oropharyngeal (or buccopharyngeal) membrane, without any contribution from the mesodermal germ layer (Figure 2). The moment in embryonic development that this membrane dissolves could be marked as the start of the mouth formation [12]. In human embryos, this phenomenon is seen between Carnegie stages (CS) 11 and 12 (±26 days post conception). Around 29 days after conception, in CS 12, ecto- and endoderm form a continuous epithelium and the oral cavity starts to develop [13].

By definition, the oropharyngeal membrane, which initially separates the stomodeum (primitive mouth) from the foregut (primitive pharynx), is a critical marker of the ecto-endodermal boundary [14]. However, the exact location of this border is controversial after onset of its rupture, primarily because this area undergoes significant developmental changes like regional growth differences and cell migration. It has been shown that the oropharyngeal membrane develops anterior to the first pharyngeal arch, and ruptures in late CS11/early CS12 [13]. In addition, the first pharyngeal arch develops lateral to the trigeminal nerve [13,15]. The mandible forms from the first pharyngeal arch, which indicates that the membrane lays anterior to the mandible [16,17]. This implies that all structures that develop posteriorly from the mandible would have to be created from foregut endoderm.

**Figure 3:**
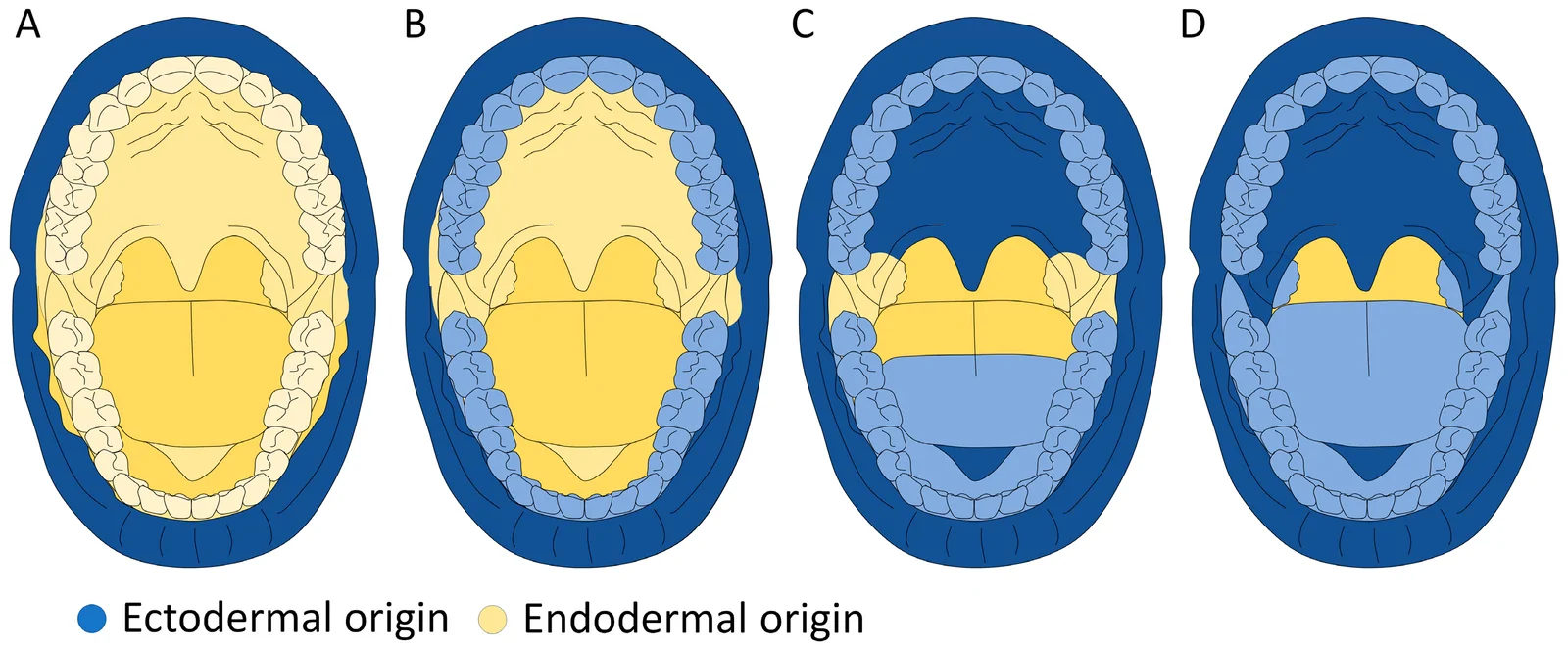
**Schematic drawing of the ecto- and endodermal origin of lining of the opened human mouth, frontal view.** The figure illustrates four current theories on the ecto- and endoderm-derived tissues in the human mouth. The blue tissue indicates an ectodermal origin, and the yellow tissue indicates an endodermal origin. (**A**) The border between ectoderm and endoderm is positioned behind the lips. (**B**) The border between ectoderm and endoderm is positioned behind the teeth. (**C**) The border between ectoderm and endoderm is positioned behind the uvula for the cranial part of the mouth, and on the posterior side of the tongue for the caudal part of the mouth. (**D**) The border between ectoderm and endoderm is positioned in the pharynx.

In the educational literature, the epithelial transition has been described as lying on the back of the tongue in the caudal part of the mouth and behind the uvula, in the pharynx for the cranial part of the mouth (Figure 3C) [18]. Other theories describe the location of this border to be posterior of the lips or teeth, or in the pharynx. In Figure 3, the four theories on the ecto-endodermal border are shown.

Knowing the exact border between ecto- and endoderm is important for a better understanding of the development of the human mouth during embryonic development. The precise border between these two germ layers might delineate the location of HPV-induced tumor initiation in the mouth. Thus, it helps to create a new approach in early-stage tumor detection after HPV infection, or in cases with a primary tumor of unknown origin.

## 2. Materials and Methods

Biomedical research article database PubMed and literature database Google Scholar were searched for studies about oral development to better understand the etiology of oral tumors. The following terms were used as inclusion criteria for this narrative review: embryonic development, mouth development, oropharyngeal membrane, buccopharyngeal membrane, salivary glands, sublingual gland, submandibular gland, parotid gland, tubarial gland, tonsils, faucial pillar, tonsillar pillar, teeth, molar, incisor, heterodontia, lamina dentalis, fungiform papillae, circumvallate papillae, foliate papillae, taste buds, ectoderm, endoderm, human papillomavirus, and oral cancer. Information about development in both humans and animals was included in our search. Articles written in a language other than English were excluded. No exclusion criteria for publication dates were used.

## 3. Results

### 3.1. Teeth

The human maxilla (upper jaw) and mandible (lower jaw) each typically contain 16 teeth, comprising 4 incisors, 2 canines, 4 premolars, and 6 molars, including the wisdom teeth.

Teeth originate from the ectodermal epithelium. This was demonstrated by endodermal cell tracing, performed by infecting Sprague-Dawley rat embryos of 9.5 days old with an adenovirus that contained endoderm-labeling lacZ [19]. The mandibulae were then cultured and X-Gal-stained to visualize the infected cells. LacZ-labeled endoderm was discovered anteriorly of the tongue, adjacent to prospective incisor epithelium, indicating that teeth develop from endoderm-neighboring ectoderm.

The aforementioned results were corroborated in 2012 [12]. Rosa26-LacZ reporter was used in Sox17-2A-iCre mice embryos around age E11 to label endoderm-derived cells. This experiment showed that the molars do not contain endoderm-derived tissue. Similarly, the incisors were not marked as endoderm-derived. As the authors claim that the second and third molar develop from the first molar (an assumption for which no evidence is found in the literature), the authors solely focused on the first molar. This tooth did not show an endodermal origin, suggesting that, based on their claim, from the first molar forward all teeth are ectoderm-derived.

In addition, Sox17-LacZ-stained Vibratome and cryostat sections were studied postnatally to investigate the origin of the third molar. The authors found that this tooth was negative for LacZ and thus does not contain any endodermal cells. They therefore concluded that all murine teeth develop from ectoderm [12].

Similar to these two studies, others also demonstrated that teeth originate from ectoderm [20]. To examine the origin of the mouth, the endoderm in chick embryos in Hamburger–Hamilton stages (HH) 8–9 (±29 h post conception) was traced. DiO (carbocyanine dye 3,3-dioctadecyl-5,5-di(4-sulfophenyl)-3,3,3,3-tetramethylindocarbocyanine) was used to label endodermal tissues. Neither the maxilla nor the oral side of the mandibular arch showed any labeled tissues, indicating an ectodermal origin. For endoderm-tracing experiments in mice, the authors used DiI staining (carbocyanine dye, further unspecified) to visualize endodermal cells. No cells were labeled in the region where the teeth develop. This study too demonstrated that oral epithelium of chick embryos and the teeth-forming regions of mice embryos are all ectoderm-derived.

In contrast to these results, the only study using ectoderm-labeling of oral tissue demonstrated that teeth develop in both ecto- and endoderm-derived tissue [21]. Identification of ectodermal cells was performed by creating transgenic axolotl embryos and analysis of sagittal sections of the oral cavity.

### 3.2. Salivary Glands

Five different salivary glands will be described: the parotids, the submandibular, the sublingual, the tubarial, and the minor salivary glands. The parotid, submandibular and sublingual salivary glands are together called the major salivary glands.

The parotid glands are bilaterally located anterior and inferior to the ear. The parotid glands start developing at 4 weeks post conception [22] and are the most common location for salivary neoplasms [23] (Figure 1).

The submandibular glands are located in the posterior portion of the submandibular triangle, which is formed by the body of the mandible superiorly, the anterior belly of the digastric muscle medially, and the posterior belly of the digastric muscle inferiorly and laterally (Figure 1). They develop from epithelium of the floor of the mouth between the fourth and 22nd week post conception [24].

The sublingual glands are positioned superior to the mylohyoid muscle and inferior to the mucosa of the floor of the mouth (Figure 1). Ducts that excrete the produced saliva into the oral cavity are of ectodermal origin [25]. Development of the sublingual glands starts laterally to the submandibular gland at six weeks post conception, after which they migrate to their final position [26]. By using Sox17-LacZ staining to trace the endodermal epithelium in Sox17-2A-iCre/R26R mice, it was demonstrated that the major glands originate from the ectoderm [12]. As no LacZ-labeled cells were found in the major salivary gland tissue, it was concluded that they are formed from ectoderm.

Hundreds of minor salivary glands are located throughout the oral cavity within the submucosa of the oral mucosa in the tissue of the buccal, labial, and lingual mucosa, the soft palate, the lateral parts of the hard palate, and the floor of the mouth (Figure 1).

To examine the origin of tongue-located salivary glands, tongues of Sox17-2A-iCre/R26R mice were analyzed by using Whole-Mount [12]. These experiments proved that the minor salivary glands on the tongue originate from endodermal epithelium. To conclude that there were no ectodermal glands located on the tongue, Alcian blue and LacZ staining were additionally performed. As this experiment showed that there was a complete overlap in the stained tissues, all of the tongue-located (minor) salivary glands originate from endoderm.

Minor salivary glands on the palate were studied by using eosin-stained sections of murine heads [12]. The glands appeared to form from both the ecto- and endodermal epithelium. The more anterior-located palatal glands originate from ectoderm, and the more posterior-located palatal glands originate from endoderm. Based on the published data, no exact border could be defined.

In 2021, additional salivary glands were described: the tubarial glands [27]. As no lineage-tracing experiment has been performed for these glands yet, the origin of this gland cannot be concluded. However, immunological experiments using various components such as Alcian blue, KRT7, KRT14, and KI67 identified a similar origin as the palatal salivary glands [28,29]. As a study on the palatal salivary glands showed that both ectoderm- and endoderm-derived salivary glands are present on the palate, we cannot yet identify the true origin of this relatively newly discovered gland [12].

### 3.3. Papillae

Papillae (taste buds) of the human mouth increase the surface area of the tongue to increase the area of contact between the tongue and the food bolus, and perceive molecules associated with tastes. There are five types of papillae: circumvallate, foliate, fungiform papillae, filiform, and incisive papillae (Figure 1). The papillae are located on both the tongue and the palate. The larger circumvallate papillae can be found on the dorsal tongue. On both sides, lateral to the circumvallate papillae, foliate papillae can be found as clefts of the tongue. Fungiform and filiform papillae are located more anteriorly, spread diffusely over the tongue. The incisive papilla is located on the anterior palate, directly behind the incisors.

Papillae originate from the endoderm [30]. This was demonstrated using DiI lineage tracing in axolotl embryos to examine their origin. The epithelium of the axolotl embryos was microinjected with DiI and counterstained to visualize endodermal tissue. Taste buds appeared to be DiI-labeled. This implies that the taste buds in axolotl embryos are of endodermal origin.

The endodermal origin of the fungiform papillae and circumvallate papillae was confirmed in mice [31]. For the analysis of fungiform papillae, parasagittal (fungiform papillae) or transverse (circumvallate papillae) sections of mosaic H253 mice were incubated in X-Gal and then red-counterstained. The staining of fungiform papillae matched the surrounding tissue, indicating not a migration but originating from the local epithelium. Based on the location of the taste buds, the fungiform papillae are not ectoderm-derived. Similar to the fungiform papillae, the circumvallate papillae-staining matched the surrounding tissue, which indicates that the circumvallate papillae are formed from endodermal epithelium [31].

Filiform papillae partially originate from the ectodermal epithelium [32]. Ectoderm marker Pax9 was studied in Pax^LacZ^ mice. Filiform papillae of Pax9-deficient mice were analyzed, and abnormal filiform papillae development was found, shown by the non-polarity of the papillae. The incomplete development of filiform papillae indicates a regulatory function of Pax9 on the morphogenesis of filiform papillae. This suggests at least a partial ectodermal origin of filiform papillae.

Others demonstrated that papillae originate from both ecto- and endoderm [12]. Sox17-LacZ and Alkaline Phosphatase (AP) were used for endoderm labeling in Sox17-2A-iCre mice. LacZ-negative/AP-positive buds indicate a non-endodermal origin of foliate trenches. The results of this experiment did not show such a pattern for the circumvallate papillae and foliate papillae, indicating that they are formed from endoderm. However, this pattern is found at the site where the filiform papillae are present. This implies that both ectoderm and endoderm are present in filiform papillae, which is in accordance with earlier research [32].

No articles studying the origin of the incisive papilla could be retrieved.

A summary of the results discussed in this review is shown in Table 1.

## 4. Discussion

Knowledge of the exact border of ectodermic as opposed to endodermic origin of oral and oropharyngeal epithelium is important as HPV-induced malignancies tend to develop from the transition areas in between these two originating linings. In this review we aimed to describe current knowledge about the origin of structures in the human mouth, aiming to give a more detailed border between ecto- and endoderm.

As ethical implications on the use of human embryos for research-restricted progress in developmental studies, embryological studies are almost always performed in animal models. Mice anatomy is more comparable to that of humans, as opposed to axolotl and chick anatomy. Therefore, in the case of disagreement between experiments, mice experiments would be the deciding factor. However, consensus between model systems was found. Different experimental techniques were used to determine the origin of the oral structures, giving a certain origin of the discussed epithelial structures.

### 4.1. Teeth

**Figure 4:**
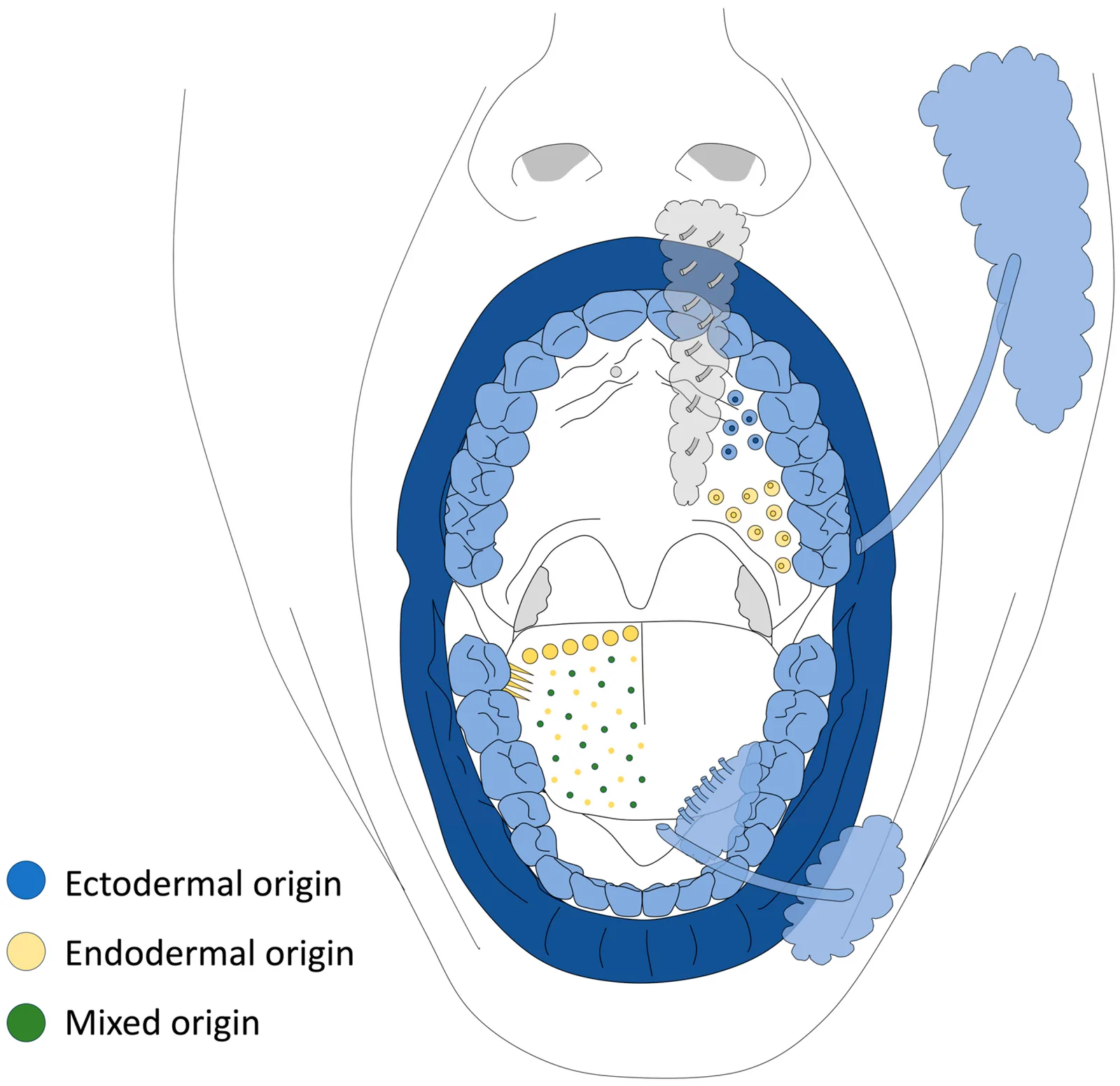
**Origin of teeth, papillae, and salivary glands.** This figure shows the origin of human teeth, papillae, and salivary glands. Ectoderm-derived tissues are shown in blue, endoderm-derived tissues are shown in yellow, tissues of mixed origin are shown in green [12,19,20,21,30,31,32].

Mammalian teeth (including incisors, canine, premolars and molars) develop from ectodermal tissue. Both developing and mature teeth were analyzed, showing the origin and development of all teeth. The origin of the teeth is illustrated in Figure 4. The tissue surrounding the teeth (e.g., the gums) is not strictly of ectodermal origin.

### 4.2. Papillae

Oral papillae originate from both ectoderm and endoderm, depending on the location of the buds. The origin of the different papillae was shown by multiple techniques. There are no substantial contradictions between the experiments. However, techniques improved over time, which could explain the more precise conclusion given in 2012 [12], compared to the conclusions of 1994 [30]. Based on their location (directly behind the incisors of the upper jaw) and the results of the other structures, we expect these papillae to be of ectodermal origin as well. The suggested probable origin of papillae is in contrast with the educational literature, in which the entire palate was claimed to be of ectodermal origin. An accurate overview of the origin of all papillae is shown in Figure 4.

### 4.3. Salivary Glands

The oral salivary glands originate from both ectoderm and endoderm, depending on the location of the gland. The ecto-endodermal transition in the oral cavity is not only located on the tongue but also on the palate (more posteriorly than the border on the tongue) as depicted in Figure 4. The origin of the salivary glands is shown in Figure 4.

The tympanic cavity and Eustachian tube to which the tubarial glands lay proximally are both derivatives of the first pharyngeal arch, consisting of endodermal tissue [33,34]. Although experiments on the origin of the tubarial glands have not been carried out, their origin can be presumed to be endodermal based on these surrounding tissues and their developmental origin [27].

Altogether, the border between ectoderm and endoderm is placed medial to the teeth, anterior of the tongue’s salivary glands, in the posterior region of the palate’s salivary glands and posterior to the filiform papillae for the papillae. For all of the discussed areas, the origins of their structures are shown in Table 2.

It is noticeable that proof of an ectodermal origin is consistently demonstrated by the lack or absence of endodermal dyes. Ectodermal dyeing has been performed only once, in axolotl. A more frequent use of ectodermal dyeing might have contributed to a decisive conclusion about the developmental origin, as non-endodermal-derived tissues could also have developed from mesoderm. Based on the developmental process and the area of interest, the involvement of mesoderm is not to be expected. An either endodermal or ectodermal origin is expected for all discussed structures. Hence, the use of ectodermal dyes in developmental research would help studying the true origin of all tissues, rather than rejecting an alternative origin.

The conclusions from the studies that were discussed in this review contradict the currently accepted theories about the ecto-endodermal border as mentioned before (Figure 3). An endodermal origin on the palate and anterior tongue is not shown in the current theories, as depicted in Figure 3C,D. However, the results of the discussed studies show endoderm-derived tissues in these regions. In this review, we demonstrated that salivary glands in the palate are of ectodermal origin, but the current theories do not correspond with these conclusions either, as depicted in Figure 3A,B. In addition to this, the major salivary glands originate from the floor of the mouth and migrate afterwards, and the major salivary glands are of ectoderm-derived tissue. It is therefore evident that the floor of the mouth is not endoderm-derived, starting dorsally to the teeth. However, as described before, teeth were found to develop in regions of both ectodermal and endodermal tissue, leaving an exact location of the transition zone undecided.

Comparison of the discussed experiments contributes to a more precise picture of mouth formation during embryonic development. The ectodermal origin of teeth that is found in this review supports the theory that is currently accepted and described as shown in the educational literature. It rejects the theory that the border between ectoderm and endoderm is positioned behind the lips, as described in Figure 3A.

Our results indicate that all theories regarding the origins of oral structures could complement each other. The papillae and salivary glands could have developed from pharyngeal endoderm and migrated to the tongue and palate. Whether this theory is correct or not, the currently accepted theory that is used in the educational literature—that the border lays behind the uvula and on the posterior tongue—does not reflect the situation in human anatomy.

The more precise transition zone as provided in our review could illustrate the predilection sites of HPV-induced cancers in the oropharyngeal region. As the border between ectoderm and endoderm is now appointed more specifically, this review contributes to a better understanding of the etiology of cancer in the oropharynx. In clinic, this will help predict the possible locations for oral carcinomas and early diagnosis and intervention. It also helps in explicitly looking for suspicious areas in the projected transition zone in case of unknown primary tumors with positive lymph nodes (the “unknown primaries”).

Future research should focus on the clarification of the origin of structures about which no studies have been published yet, namely, the tonsils, the tubarial glands and faucial pillars. As the occasion of this review was to identify the location of oral primary tumors and the site of these structures is thus clinically relevant, their developmental origin might play an important role in understanding the development of the pathology and early diagnosis. However, genetic expression patterns play a key role in understanding tumor initiation after HPV infections [35]. In addition, further research could focus on the use of stembroid models to investigate the exact origin of all structures in the human oral cavity [36]. After that, genetic regulation after HPV infection could be studied to understand cell behavior prior to and after HPV infection [37].

This review demonstrated the origin of several structures in the human oral cavity that develop during the embryonic stage. An overview of the current studies was provided to verify currently accepted theories about the formation of the oral cavity in humans. A contrast with the ectoderm–endoderm transition zone as described in the literature was found. A new theory was created and can be used as a reference for developing structures in the oral cavity.

## Figures and Tables

**Table 1:** Sources of used studies in the field of embryonic origin of oral and oropharyngeal tissues, with key findings.

Authors	Oral or Oropharyngeal Structure	Claimed Origin
Barlow & Northcutt (1994) [30]	Papillae	Endodermal
Imai et al. (1998) [19]	Teeth	Ectodermal
Jonker et al. (2004) [32]	Filiform papillae	Partially ectodermal
Ohazama et al. (2010) [20]	Teeth	Ectodermal
Rothova et al. (2012) [12]	Teeth	Ectodermal
	Major salivary glands	Ectodermal
	Tongue-located salivary glands	Endodermal
	Palate-located salivary glands	Ecto- and endodermal
	Foliate trenches	Non-endodermal
	Circumvallate papillae	Endodermal
	Foliate papillae	Endodermal
	Filiform papillae	Ecto- and endodermal
Soukup et al. (2013) [10]	Teeth	Ecto- and endodermal
Stone et al. (1995) [31]	Fungiform papillae	Endodermal
	Circumvallate papillae	Endodermal

**Table 2:** **Embryonic origin of oral structures** discussed in this review, with their corresponding origins.

Ectodermal Origin	Endodermal Origin	Mixed Origin
Teeth	Circumvallate papillae	Filiform papillae
Parotid glands and ducts	Foliate papillae	Faucial pillars *
Sublingual glands	Fungiform papillae	
Submandibular glands	Salivary glands of tongue	
Anterior palatal salivary glands	Posterior palatal salivary glands	
Incisive papilla *	Tonsils *	
	Tubarial glands *	

Note: Only the epithelial lining of the structures mentioned in this table is of either ectodermal or endodermal origin. Their supporting tissues generally have a mesodermal/mesenchymal origin. * These structures were placed in this table without direct literature-based evidence but were based on their anatomical location relative to adjacent structures with established embryonic origins.

## Data Availability

No new data were created or analyzed in this study. Data sharing is not applicable to this article.
